# Mesenchymal Stem Cells as Vectors for Lung Cancer Therapy

**DOI:** 10.1159/000351284

**Published:** 2013-05-23

**Authors:** Krishna K. Kolluri, Geoff J. Laurent, Sam M. Janes

**Affiliations:** ^a^Lungs for Living Research Centre, University College London, London, UK; ^b^Centre for Cell Therapy and Regenerative Medicine, University of Western Australia, Perth, W.A., Australia

**Keywords:** Apoptosis, Delivery vectors, Homing, Lung cancer, Mesenchymal stem cells, TRAIL

## Abstract

Despite recent advances in treatment, lung cancer accounts for one third of all cancer-related deaths, underlining the need of development of new therapies. Mesenchymal stem cells (MSCs) possess the ability to specifically home into tumours and their metastases. This property of MSCs could be exploited for the delivery of various anti-tumour agents directly into tumours. However, MSCs are not simple delivery vehicles but cells with active physiological process. This review outlines various agents which can be delivered by MSCs with substantial emphasis on TRAIL (tumour necrosis factor-related apoptosis-inducing ligand).

## Introduction

Lung cancer is one of the leading causes of mortality and morbidity in the world and accounts for one third of all the cancer-related deaths [1]. An estimated 1.61 million people across the world were diagnosed with lung cancer in 2008 [2]. Non-small cell lung cancer accounts for 80% of all the lung cancer cases and its 5-year survival remains 8-15% [3]. Current treatments of lung cancer include surgery, radiotherapy and chemotherapy. For metastatic lung cancer, chemotherapy with the combination of cisplatin and pemetrexed is used as first-line treatment. EGFR antagonists like erlotinib and gefitinib are recommended in the low percentage of cancers with EGFR-tyrosine kinase mutations. Despite the introduction of new therapies, lung cancer kills more people than breast, colon and prostate cancers combined, and there has been little overall improvement in patient survival in 3 decades [4]. This justifies the need for new and innovative therapies. Stem cells may be able to deliver such therapies to the site of tumours with minimal adverse effects.

## Mesenchymal Stem Cells

Mesenchymal stem cells (MSCs) are a type of bone marrow-derived stem cell, which can differentiate in vitro into osteoblasts, chondrocytes and adipocytes. They do not possess any unique markers for their identification, so their identification relies on the expression of CD73, CD90 and CD105 while lacking CD34, CD45 and other haematopoietic stem cell markers [5].

MSCs lack the expression of MHC II and its co-stimulatory molecules CD80 and CD86 and CD40 [6]. This low immunogenicity of MSCs may make allogeneic cells incapable of eliciting an immune response when used in immunocompetent patients hence avoiding the need for human leucocyte antigen matching and allowing an off-the-shelf therapy [7]. This paves the way for using MSCs as cell-based therapeutic vectors for the treatment of cancers. Indeed, clinical trials using MSCs for treatment of a wide variety of diseases including graft-versus-host disease and Crohn's disease have proved delivery of allogeneic MSCs is safe. MSCs are also easily extracted and readily expandable with up to 50 population doublings in 10 weeks [8]. Taken together, these properties may enable the creation of MSC cell banks.

## MSC Homing to Tumours and Mediators Involved

It has been widely demonstrated that MSCs home to and infiltrate into areas of new stroma formation possibly forming crucial stromal support [9]. This has been shown in several models, including lung metastases [10,11], Kaposi sarcomas [12] and gliomas [13]. However, their role once integrated within the tumour environment is unknown.

The precise mechanism of homing of MSCs to the tumours is not fully mapped, but it was widely accepted that the chemokines released by the tumours attract MSCs. This is substantiated by the presence of a wide variety of chemokine receptors on the MSC cell surface and experiments in vitro and in mouse models that have either over- or under-expressed these receptors, showing a change in MSC homing capabilities [14,15,16,17,18]. There are several different ligands and receptors postulated to play a role in MSC migration. However, there is general agreement that these studies have not yet been able to pinpoint the exact chemokine and its respective receptor that governs MSC tumour tropism, and there may indeed be a combination of receptors and chemokines responsible.

CXCL12 and its receptor CXCR4 have generated particular interest in MSC homing. Their knockouts are universally fatal in utero and their role in migration of haematopoietic cell migration is well characterised [19,20]. Several tumours are known to release CXCL12 [21,22] and studies show over-expression of these receptors leads to increased MSCs migration to infarcted myocardium [23]. However, knockdown of these receptors does not mitigate MSC homing capability [24]. This can be interpreted that the CXCL12 ligand and its receptor CXCR4 might be capable of inducing some MSC migration but they are not the only receptors responsible for MSC homing. This is further substantiated by the fact that some MSCs do not express this receptor at all [18].

Work on MSC homing is complicated and varying results may be explained by a number of factors. MSCs are extracted from various tissues and their lack of unique identification markers to classify them results in a number of different populations being used making cross-referencing results difficult. Furthermore, different in vitro culture conditions and the passage numbers used alter the expression of cell surface receptors [16,25]. This results in lack of homogeneity of MSCs being used in laboratories likely explaining the variability seen. Taken together, MSC migration is highly likely to be dependent on the expression of a number of chemokine receptors on their cell surface [26,27].

## MSCs as Delivery Vectors for Pro-Apoptotic Agents

The homing capability of MSC can be exploited to deliver pro-apoptotic agents straight into the tumour micro-environment. Several studies have achieved this with varying success and are summarised in table 1.

The majority of studies have used MSCs engineered to express and deliver a variety of cytokines. Interleukin (IL)-2, an immune modulatory cytokine, has been shown when over-expressed by MSCs to improve immune surveillance against tumours and reduce metastasis from a subcutaneous model [28]. Similarly CX3CL1, a chemokine which activates both T cells and NK cells when delivered by MSCs, leads to a substantial decrease in lung tumours induced by intravenous delivery of melanoma cells [29]. Interferon-β, which induces differentiation and S-phase accumulation leading to apoptosis, when expressed by genetically engineered MSCs suppresses pancreatic tumours, prostate cancers, breast cancers and melanomas in animal models [30,31,32,33]. Finally, a similar effect occurs with the delivery of IL-12-expressing MSC in renal cell carcinoma [34].

An exciting set of viruses which selectively target and inhibit tumour cells without affecting normal cells are termed oncolytic viruses [35]. These viruses are genetically engineered to selectively infect and destroy tumour cells. However, their delivery to the tumour site remains a major challenge [36]. Using MSC tumour tropism raises a new possible modality of virus delivery. MSCs would again act as carrier vectors for the oncolytic virus and this delivery mechanism comes with the added advantage of limiting the recipient immune response to the virus to a minimum. This technique has been very successfully used in several tumour models, which include breast and lung metastases [37,][38] and ovarian cancer [39]. Indeed, a recent study has demonstrated the feasibility of treating ovarian cancer using MSC oncolytic virus, paving the way for a phase I clinical trial [40].

MSCs have also been engineered to express an enzyme which converts a pro-drug into a cytotoxic agent at the site of tumours. This has been successfully demonstrated in a glioma model [41] where MSCs were engineered to express the herpes simplex virus-thymidine kinase which converts the prodrug ganciclovir at the tumour site. However, this approach may be limited by the toxicity to the carrier MSCs. A similar approach has been used to convert 5-fluorocytosine to 5-fluorouracil by MSCs expressing cytosine deaminase enzyme in melanoma [42] and colon cancer models [43]. MSCs have also been genetically modified to express rabbit carboxylesterase enzyme, which can efficiently convert the prodrug CPT-11 into the active drug SN-38, which acts as a potent topoisomerase I inhibitor [44]. In a different approach, nano-sized exosomes which are mass produced by MSC [45] have been extracted and used to deliver a variety of therapeutics including siRNA [46].

There is increasing interest in the use of nanoparticles in a variety of biomedical applications. However, the ability to deliver them efficiently to a disease that is systemically distributed remains a key challenge. Again, MSC tumour tropism has been used as a method of tumour targeting. A silica nanorattle-doxorubicin drug delivery system was efficiently anchored to MSCs by specific antibody targeting the CD90 receptor on the MSC cell surface and successfully delivered into a glioma model [47]. Interest has also arisen in using nanoparticles as a method of tracking MSC homing to tumours. Iron oxide nanoparticles phagocytized by MSCs have been used to identify MSC homing to pulmonary lung metastases using magnetic resonance imaging [11].

## Delivery of TRAIL

**Fig. 1:**
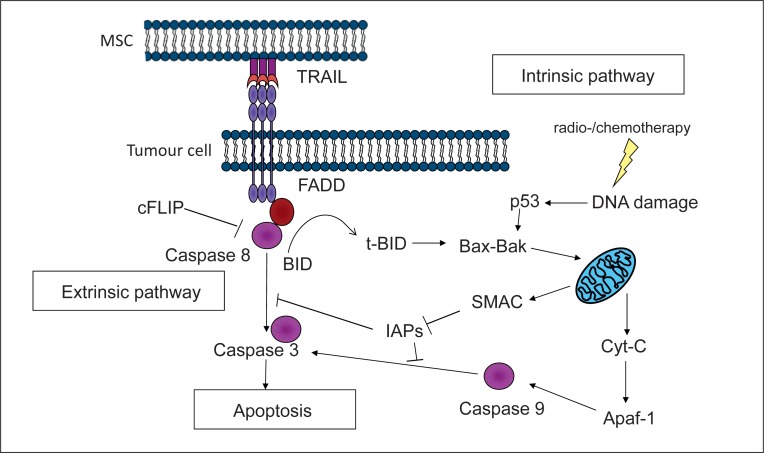
TRAIL signalling induces the extrinsic apoptotic pathway. TRAIL triggers the extrinsic apoptotic pathway while conventional chemotherapeutics and radiotherapy trigger the intrinsic apoptotic pathway mediated by mitochondria. There is crosstalk between the two pathways mediated by cleavage of BID into t-BID by caspase 8. cFLIP and IAPs are potent inhibitors of apoptotic proteins and their inhibition could induce synergistic effects by simultaneous triggering of both pathways. FADD = FAS-activated death domain; BID = BH3 interacting-domain death agonist; BAK = Bcl-2 homologous antagonist; Cyt-C = cytochrome c; Apaf-1 = apoptotic protease-activating factor 1.

TRAIL (tumour necrosis factor-related apoptosis-inducing ligand) is the most studied and well-characterised pro-apoptotic agent widely accepted to be ideal as MSC cargo. TRAIL, also known as APO2 ligand, is a type II transmembrane protein with 281 amino acids and a member of the TNF death ligand superfamily. TRAIL triggers the extrinsic death pathway (fig. 1). The physiological function of TRAIL is not fully understood. However, it is believed to play a role in the control of auto-reactive immune cells and immune surveillance especially against transformed cells [48].

There are five types of TRAIL receptors identified to date and they include TRAIL-R1, TRAIL-R2, TRAIL-R3 and TRAIL-R4. However, only TRAIL-R1 (death receptor-4, DR-4) and TRAIL-R2 (DR-5) are able to transduce a signal into the cell after binding of TRAIL to their extracellular domains. The receptors TRAIL-R3 and TRAIL-R4 do possess extracellular domains capable of binding to the ligand but lack an intracellular cytoplasmic domain, thus failing to mediate death signals to the intracellular apoptotic machinery. Hence, TRAIL-R3 and TRAIL-R4 act as decoy receptors that antagonize TRAIL-induced apoptosis. All these receptors form heterotrimers upon binding of the ligand. Osteoprotegerin is a soluble protein which possesses the capability of binding to TRAIL with low affinity [49]. This protein is not expressed on the cell surface.

**Fig. 2:**
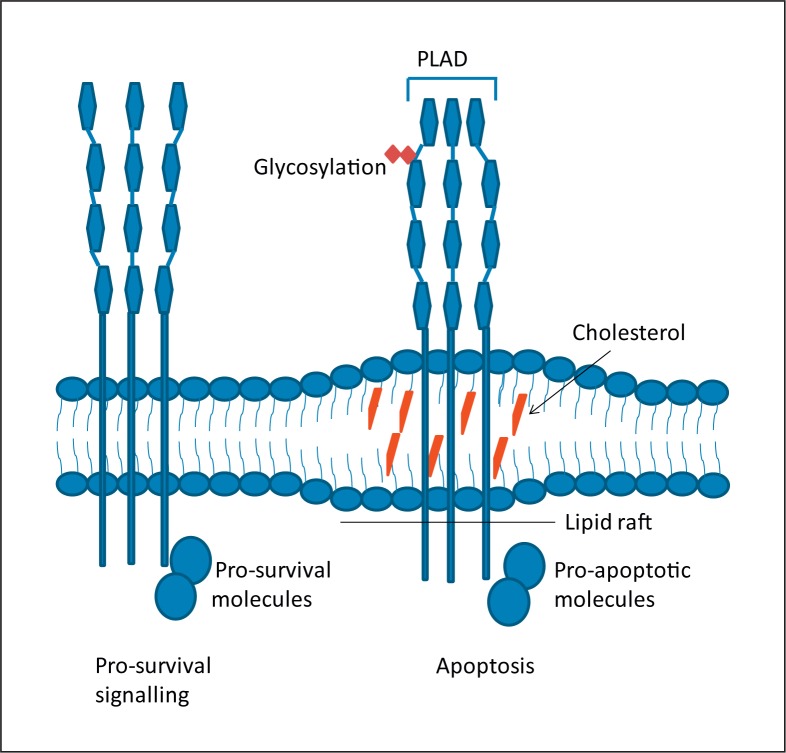
TRAIL signalling varies based on death receptor location and glycosylation. TRAIL induces apoptosis pathways when the death receptors are glycosylated or forms pre-ligand binding assembly domain (PLAD) or when located on lipid rafts.

The novelty of TRAIL is that it only induces apoptosis in transformed cells with virtually no effect on normal cells. This makes TRAIL a unique therapeutic with very few off-target adverse effects characteristic of chemotherapeutic agents and radiation. The mechanism for this selective targeting of tumour cells is not well characterised. The decoy receptor theory states that the normal cells express decoy receptors while the transformation of cells makes them express DR4 and DR5 thus making them vulnerable to TRAIL [50]. However, this theory is not widely accepted and it is believed that the selective cytotoxicity of TRAIL occurs beneath the cell membrane. One intriguing study shows that in tumours TRAIL receptors lead to apoptosis when expressed within lipid rafts of the cell membrane (fig. 2). These rafts concentrate pro-apoptotic downstream signalling molecules internally. In normal tissue, however, the receptors are largely in the non-raft areas and TRAIL binding can lead to pro-survival pathway activation [51]. Other studies have identified the TRAIL receptor glycosylation status [52] and pre-ligand binding assembly domain of receptors playing roles in TRAIL sensitivity [53]. It is likely that TRAIL sensitivity is multi-factorial and cannot be ascribed to any single mechanism.

The selective tumour-specific cytotoxicity of TRAIL has led to hailing it as a ‘silver bullet’ for the treatment of cancer. However, its limited bioavailability and poor pharmacokinetic profile have made its use a serious challenge. The half-life of TRAIL is very short at around 30 min [54]. To circumvent this problem, we and others have engineered MSC to constitutively express TRAIL. This has been demonstrated effective in several models, including glioma [55], pancreatic cancer [56] and a lung metastasis model [43]. MSC-TRAIL cells home into the tumours and expresses TRAIL leading to selective apoptosis of tumour cells with no detectable cytotoxicity to the surrounding tissue. We have used a Tet-On promoter system allowing the controlled release of TRAIL with the addition of doxycycline [10]. Interestingly, the tumour killing capability of MSCs expressing TRAIL is significantly higher than that of recombinant TRAIL [10].

However, not all tumours are fully sensitive to TRAIL. TRAIL triggers the extrinsic apoptotic pathway, while conventional chemotherapeutics and radiation trigger the intrinsic apoptotic pathway (fig. 2). It would be ideal to trigger the simultaneous activation of both pathways to harness synergistic effects. There is known synergy between traditional chemotherapy agents and TRAIL. This synergy results in increased apoptosis by amplification of apoptotic signals through crosstalk between the two apoptotic pathways [57]. A number of chemotherapeutic agents have demonstrated synergy both in vitro and in vivo: cisplatin [58], vorinostat [59], pemetrexed [60], sunitinib [61], etoposide [62], doxorubicin [62] and bortezomib [63]. Furthermore, the combination of chemotherapeutics and MSC expressing TRAIL was shown to be synergistic with bortezomib [64] in myeloma cells and vorinostat in lung cancer [65].

Identifying and targeting proteins responsible for TRAIL resistance may also increase the anti-tumour potency of TRAIL, such as cFLIP (cellular FLICE-inhibitory protein) [66,67], cIAP1/cIAP2 [68] and XIAP [56,69,70]. Another potential use of MSC-delivered TRAIL would be to decrease the required dosage of chemotherapeutic agents improving drug tolerance and reducing adverse effects [71].

## Immunosuppressive Effects of MSCs

The ability of MSCs to home effectively to tumours makes them an attractive therapeutic option. However, MSCs are not merely vehicles which transport the therapies but are cells possessing physiological properties.

It is widely accepted that in large numbers MSCs possess immunosuppressive effects in vitro. They are capable of arresting the immune cells in G0/G1 phases thus preventing the S-phase entry and subsequent cell division. This has been demonstrated in T cells [72], B cells [73] and dendritic cells [74]. This leads to reduced cytotoxic capability of T cells and antibody production of B cells. MSCs also exert an immunosuppressive effect by activation of regulatory T cells. These properties of MSCs are clinically exploited for the treatment of graft-versus-host disease after bone marrow transplantation [75].

These anti-inflammatory effects have been tested in a number of clinical trials in inflammatory conditions, such as inflammatory bowel disease and chronic obstructive pulmonary disease, and in other diseases, such as cardiac disease. Several studies show an enhanced cardiac function [76,77,78,79] and reduced infarct size [78] by injecting MSCs after myocardial infarction and chronic ischemic heart failure. The exact mechanisms of this effect are to be characterised but have been attributed to anti-inflammatory properties of MSCs. These anti-inflammatory properties have also been demonstrated in murine models of pulmonary fibrosis and acute lung injury, and were thought the result of the paracrine effect of secreted tropic factors [80]. The Battacharya laboratory has also shown that MSCs can protect against acute lung injury by donating their mitochondria to alveolar epithelial cells [81].

The immunosuppressive capability of MSCs could, however, be a double-edged sword. Their immunosuppressive nature could potentially interfere with any physiological anti-cancer immune cell function in the tumour environment.

## Direct Effects of MSCs on Tumour Biology

Reports on untransduced MSC effects on tumour growth are mixed. The majority of work suggests MSCs not only home to tumours but also have intrinsic anti-tumour properties. MSCs alone lead to benefit in a murine glioma model [82], in our studies of pulmonary metastases [6] and in a breast cancer metastasis model with either intravenous or intra-tumour delivery of MSCs significantly reducing the growth and metastasis [83]. The mechanism of this anti-tumour effect is not fully established, although MSCs have been shown to down-regulate many pro-survival genes, such as AKT in the Kaposi sarcoma mouse model [12] and NF-κB in hepatoma and breast cancer cells [84].

In specific context, however, MSCs can appear to be tumour promoting, which was demonstrated by tumour development after subcutaneous co-administration of MSCs with allogeneic melanoma cells producing tumours, while allogeneic melanoma cells seeded on their own are not capable of tumour induction [85]. This effect was attributed to the immunosuppressive effect of MSCs which suppressed the host immune reaction to the allogeneic melanoma cells.

**Fig. 3:**
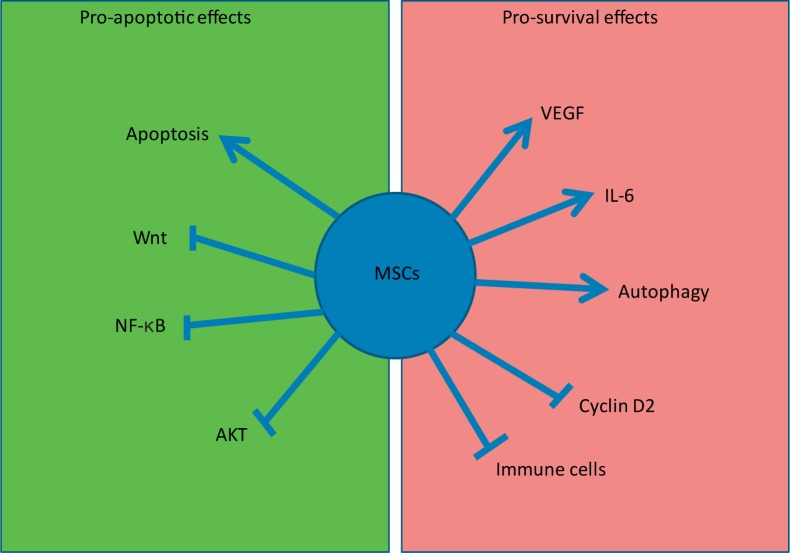
MSCs are not inert carriers. MSCs exert both pro-survival and pro-apoptotic effects on tumours. Their pro-apoptotic effects include inhibition of Akt, Wnt and NF-κB signalling. MSCs themselves induce apoptosis in some tumours. They also exert pro-survival effects by inducing VEGF and STAT3 activation. They suppress immune cells thereby reducing immune surveillance of tumours. They undergo autophagy and release pro-survival paracrine factors. They inhibit cyclin D2.

As discussed earlier, MSCs do produce a wide array of chemokines, cytokines and growth factors (fig. 3). They may also produce and secrete growth factor signalling that promotes survival in tumour cells resulting in enhanced tumour burden and metastases. It has been demonstrated that MSCs enhance the in vivo growth of Burkett's lymphoma cells through a VEGF-dependent mechanism [86]. The growth of breast cancer cells was augmented by IL-6 secreted by MSCs via STAT3 activation [87,88]. It has also been shown that MSCs can down-regulate cyclin D2 and arrest chronic myeloid leukaemia cells in G0/G1 phase preserving their proliferative capacity and reducing apoptosis in vivo [74]. Furthermore, under the nutrient-depleted conditions of the tumour micro-environment, MSCs utilize autophagy for survival and secrete anti-apoptotic factors that facilitate solid tumour survival and growth in breast cancer cells [89].

Another important and serious concern is the ambiguity that MSCs might themselves undergo malignant transformation. Karyotype abnormalities have been noticed after in vitro passage of murine MSCs [90,91,92] and transformations of bone marrow-derived cells have been implicated in a murine gastric carcinoma model [93]. Human MSCs, however, have stable karyotypes in culture and exhibit senescence with features of shortening telomeres over a 44-week culture period [94]. There have been about 300 clinical trials in clinicaltrials.gov injecting MSCs for cell therapy with no reported incident of MSC malignant transformation.

## From Bench to Bedside

Developing a cellular therapy using MSCs as delivery vectors is the ultimate goal of this area of research and MSCs have exhibited the potential for clinical translation. MSCs can be easily isolated, cultured in flasks and genetically modified. Their allogeneic application confers them an added advantage of being a possible off-the-shelf therapeutic. Their ability to target metastasis and provide a local high concentration of their cargo makes them unique.

Many phase I and II clinical trials involving MSCs for a variety of treatments were recorded in the largest clinical trial database, clinicaltrials.gov. The therapeutic areas include graft-versus-host disease, ischaemic cardiac disease, Crohn's disease and chronic obstructive pulmonary disease [95]. However, there are no reported trials of the use of MSC as delivery agents for anti-tumour therapy to date.

## Conclusion

MSC have the potential to be ideal delivery vectors for a variety of pro-apoptotic agents in treating cancers. The lack of knowledge of MSC physiology within the tumour environment is producing caution, and more robust studies characterising their homing mechanisms may improve proposed therapies. Indeed, simple questions, such as how many cells need to be given and when, remain unanswered. However, their role as an adjunct in patients with metastatic tumours looks hopeful.

## Financial Disclosure and Conflicts of Interest

All authors declare no conflicts of interest.

## Figures and Tables

**Table 1:** Anti-tumour agents delivered by MSCs

Agent	Rationale	Model	References
IL-2	immune modulatory	subcutaneous model	28

CX3CL	activates T cells and NK cells	melanoma lung metastasis	29

Interferon-β	induces differentiation and S-phase arrest	pancreatic cancer prostate cancer breast cancer melanoma	30 – 34

IL-12	activates T cells and NK cells	renal cell carcinoma	34

Oncolytic virus	destroys tumours by viral infection	breast cancer lung cancer ovarian cancer lung metastasis	37 – 39

HSV-tk	conversion of ganciclovir to active cytotoxic drugs	glioma	41

Cytosine deaminase	converts 5-fluorocytosine to 5-fluorouracil	melanoma colon cancer	42, 43

rCE	converts the pro-drug CPT-11 to SN-38, a potent topo-isomerase I inhibitor	glioma	44

Nanoparticle	silica nanorattle-doxorubicin	glioma	47

TRAIL	tumour-specific death ligand	glioma pancreatic cancer lung metastasis	10, 54, 55

MSCs have been used to deliver a variety of anti-tumour agents. The rationale behind their use and the models used are described with references. HSV-tk = Herpes simplex virus thymidine kinase; rCE = rabbit carboxylesterase enzyme.
